# Endotoxin activity assay and polymyxin B hemoperfusion use in a cohort of critically ill patients

**DOI:** 10.1186/cc13598

**Published:** 2014-03-17

**Authors:** SL Cutuli, G De Pascale, V Alicino, S Cicconi, V Di Gravio, D Silvestri, D Giacobelli, E Gasperin, S Marsili, MS Vallecoccia, M Antonelli

**Affiliations:** 1 Sacro Cuore Catholic University, Rome, Italy

## Introduction

Endotoxin plays a crucial role in the pathogenesis of severe sepsis and septic shock (SS&SSh) [1]. The aim of this study is to analyze the impact of extracorporeal endotoxin removal with polymyxin B hemoperfusion (PMX-DHP) (Toraymyxin^®^).

## Methods

All patients admitted to our ICU between 1 April 2011 and 30 June 2013 who developed SS&SSh and underwent endotoxin activity assay (EAA) measurement were retrospectively evaluated.

## Results

During the study period, EAA was dosed in 100 patients. Eighty- one patients were affected by septic shock. The source of infection was identified in 70.4% of cases (45% abdominal) and the percentage of microbiologically confirmed episodes was 77% (81% Gram-negatives). The mortality rate was 49%. The mean EAA level was 0.66 ± 0.2, and in 66% of patients the value was higher than 0.6. No significant differences were found in terms of SAPS II (*P *= 0.32) and SOFA score (*P *= 0.67), according to EAA level (>/≤0.6). Patients with levels >0.6 presented a higher percentage of microbiologically confirmed infections (84% vs. 66%; *P *= 0.09). Thirty-two of 66 patients with EAA >0.6 were treated with Toraymyxin^®^. No complications leading to treatment interruption were recorded and a relevant decrease of cardiovascular SOFA score and lactate levels were observed 72 hours after treatment (*P *= 0.05 and *P *= 0.06, respectively). Source control and Toraymyxin^® ^treatment resulted as the only modifiable factors improving the ICU survival rate (Table 1).

**Table 1:** Multivariate analysis for ICU mortality risk factors

	*P *value	OR (95% CI)
SAPS II score	0.04	1.1 (1.01 to 1.1;
Septic shock	0.02	10.4 (1.5 to 71)
PMX-DHP	0.04	0.2 (0.1 to 0.9)
Source control	0.01	0.1 (0.03 to 0.5)

## Conclusion

EAA is a rapid and reliable method to identify patients who may be treated with polymyxin B hemoperfusion. Source control and extracorporeal endotoxin removal have appeared as two effective interventions that should be implemented in the early management of patients with SS&SSh.
